# Anesthetic Management of Laparoscopic Pyloromyotomy for Pyloric Stenosis in a Neonate with Hereditary Spherocytosis

**DOI:** 10.7759/cureus.7277

**Published:** 2020-03-15

**Authors:** Akshatha S Kamath, Minal Joshi, Kimmy Bais, Uday Patil, Joel Yarmush

**Affiliations:** 1 Anesthesiology, NewYork-Presbyterian Brooklyn Methodist Hospital, Brooklyn, USA; 2 Pediatrics/Neonatal Perinatal Medicine, Icahn School of Medicine at Mt. Sinai and New York City Health + Hospitals, Elmhurst, USA

**Keywords:** hyperbilirubinemia, hereditary spherocytosis, hemolytic anemia, pyloric stenosis, neonate

## Abstract

We describe a case of hereditary spherocytosis in a neonate with pyloric stenosis requiring laparoscopic pyloromyotomy. Hereditary spherocytosis is the most commonly inherited hemolytic anemia causing hyperbilirubinemia and mild anemia. Anesthetic management for laparoscopic pyloromyotomy is challenging. Multiple factors involved, such as anemia, hyperbilirubinemia, and the effect of drugs, play an important role in anesthetic management.

## Introduction

Anesthesia for neonates can be challenging due to their unique physiology, especially with significant medical conditions. It is crucial to anticipate and prevent complications while planning anesthesia in neonates with multiple comorbidities. In the northern European population, with an incidence of 1:2000, hereditary spherocytosis is the most common inherited hemolytic anemia [1]. Infantile pyloric stenosis has an incidence of 2-3.5 per 1000 live births. We report a case of a neonate with hereditary spherocytosis, hyperbilirubinemia, and mild anemia who presented to us with hypertrophic pyloric stenosis for laparoscopic pyloromyotomy.

## Case presentation

**Figure 1:**
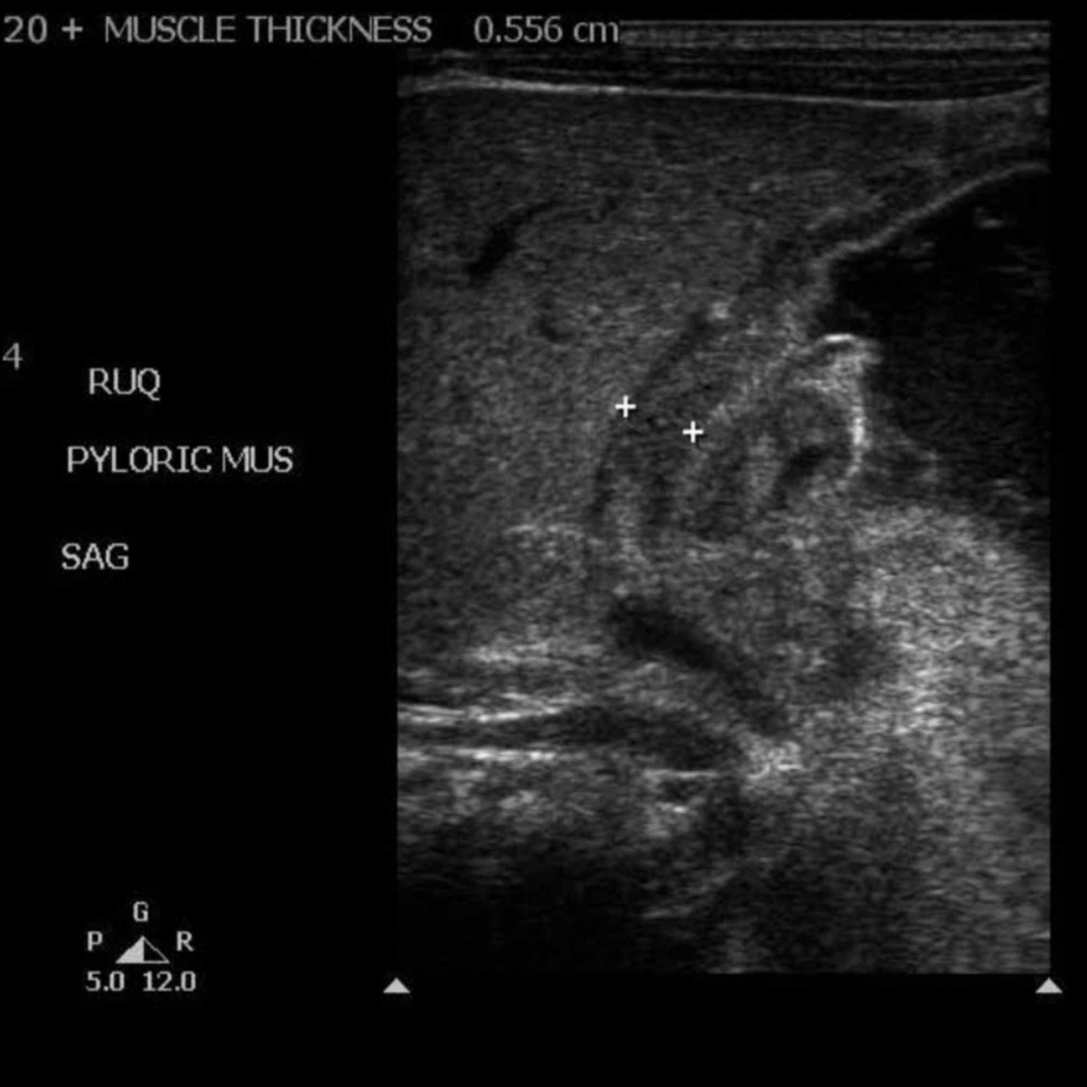
Ultrasound image showing the ingested material in the gastric antrum and pyloric stenosis US: ultrasound; SAG: sagittal plane; pyloric mus: pyloric muscle; RUQ: right upper quadrant

A three-week-old term male weighing 3860 gm with a history of hereditary spherocytosis, hyperbilirubinemia, and mild anemia presented with projectile vomiting for 24 hours. An ultrasound showed features consistent with pyloric stenosis (Figure 1). The patient had milk approximately four hours prior to the examination and the stomach remained distended with ingested material. His acid-base balance was corrected by hydration and potassium replacement. Hemoglobin was low, 7.5 g/dl /hematocrit 20%, which was corrected by red cell transfusion. Venous blood gas showed no alkalosis on the morning of surgery, and the rest of the lab values are documented in Table 1.

**Table 1:** Venous blood gas and labs

PH	7.45
PCO_2_	39mmHg
PO_2_	37mmHg
Base excess	3mmol/L
Hemoglobin	9.9mg/dl
Hematocrit	29%
White blood count	8.8/ml
Glucose	89mg/dl
Potassium levels	4.2mmol/liter
Chloride	101mmol/L
Bicarbonate	27mmol/L
Aspartate aminotransferase	31U/L
Alanine transaminase	39U/L
Bilirubin	13.9mg/dL

In the operating room, he was placed on a warm underbody blanket and standard American Society of Anesthesiologists (ASA) monitors were applied. An oro-gastric tube was placed and adequately suctioned in the lateral, supine, and prone positions to empty the stomach contents. After adequate preoxygenation, the anesthesiologist intubated the trachea after induction with propofol successfully on the first attempt. Antibiotic intravenous (IV) was administered for surgical prophylaxis. Pressure control ventilation was done and anesthesia was maintained with sevoflurane and carefully titrated fentanyl IV. The procedure was uneventful and the vital signs were maintained within normal limits. The surgeon infiltrated the skin with local anesthetic for the postoperative analgesia. We did not administer acetaminophen to avoid an exacerbating hepatic injury if any. At the end of the procedure, the patient was extubated after the adequate return of spontaneous breathing. He was monitored in the pediatric intensive care unit (PICU). He was placed on intravenous hydration, over the next 12 hours gradually re-introduced feeds and was doing well and was hence discharged home.

## Discussion

Hereditary spherocytosis disorder is inherited mostly in an autosomal dominant fashion [2]. Causative factors are the gene mutations affecting the formation of cytoskeletal proteins, such as spectrin, ankyrin, band 4.2, band 3, which promotes the formation of spherocytes [3]. These red cells, aka spherocytes, are osmotically fragile and inelastic, leading to short survival. In the cases that have been studied, the severity of hereditary spherocytosis is correlated to the loss of spectrin [4]. Thus, they are prone to break down in the spleen. Jaundice and pigment gall stones are commonly seen, secondary to chronic hemolysis. This may be accompanied by splenomegaly in older children. Unconjugated bilirubin can cross the blood-brain barrier, could be toxic to neurons, and can cause apoptosis and necrosis of the neurons [5]. The risk of bilirubin-induced neurologic dysfunction is high in term and preterm neonates when the total bilirubin concentration is ≥ 25 mg/dL. Patients with hereditary spherocytosis are at risk for an aplastic crisis secondary to parvovirus infection, which could be fatal.

Our patient had moderate hereditary spherocytosis as per the hereditary spherocytosis classification, with a reticulocyte count of 6.4%, hemoglobin 9 g/dl at birth, and unconjugated bilirubin of 13 g/dl [6]. Important concerns are to treat the complications of severe hemolysis, chronic anemia, and their consequent complications. Prolonged fasting, as well as a longer surgery duration, can precipitate a rise in bilirubin levels. Hence, it’s essential to correct the dehydration prior to the surgery. Hyperbilirubinemia should be treated preoperatively with direct photo light therapy, and if severe, with exchange transfusions. Blood transfusions should be given to correct anemia secondary to ongoing hemolysis. High bilirubin levels and blood transfusions may further lead to benign postoperative intrahepatic cholestasis, which is associated with high alkaline phosphatase and normal transaminase levels. These patients may have associated renal dysfunction secondary to hemolysis. Sulfa drugs, like thiazide diuretics, furosemide, and barbiturates, can cause oxidative injury to the cells are to be avoided [7]. Conditions like alkalosis and hypothermia could lead to the left shift of the oxygen dissociation curve, which should be avoided. It’s necessary to avoid hypoxia and hypotension, which can reduce tissue oxygen delivery [8]. Narcotics depress the respiratory centers and shift the CO_2_ response curve to the right, needing higher CO_2_ levels to stimulate breathing. Inhaled anesthetics, barbiturates, and muscle relaxants are acceptable choices.

Pyloric stenosis is commonly seen in three- to five-week-old males presenting with projectile nonbilious vomiting. The risk factors implicated to cause pyloric stenosis are maternal smoking, history of premature birth, and macrolide antibiotics use before two weeks of age, genetic factors, and formula feeding [9-10]. The infant is generally dehydrated and may have hypokalemic hypochloremic metabolic alkalosis with paradoxical aciduria. Owing to the gastric outlet obstruction, the risk of hypoxemia secondary to pulmonary aspiration and the resulting postoperative pulmonary complications is substantial [11]. Metabolic alkalosis can reduce oxygen delivery to the tissues by shifting the oxygen dissociation curve to the left and increase the risk of postoperative apnea, it’s advised to optimize the acid-base status preoperatively and bring the serum bicarbonate levels to less than <30 mEq/L [12]. Chloride levels of 100 mEq/L in the baby who is urinating show a good intravascular volume status and that he can safely undergo the surgical procedure. As per a study by Cook et al., suctioning the stomach with a wide bore catheter helps get rid of 98% of the stomach contents [13]. A study showed that inhalation induction could be safely carried out if the stomach contents are adequately suctioned prior to induction [14]. However, it would be ideal to confirm gastric emptying after suction with an on-table ultrasound examination.

In the above case scenario, we had a three-week-old baby with problems of anemia, hyperbilirubinemia and full stomach presenting for laparoscopic pyloromyotomy. Preoperative hemoglobin target needs to be individualized, as anemia can lead to tachycardia, poor weight gain, increased requirement of supplemental oxygen, or increased frequency of apnea or bradycardia. Phototherapy should be used for prolonged surgery. Prolonged fasting should be avoided, as it can cause hyperbilirubinemia (hereditary spherocytosis can co-exist with Gilbert’s syndrome). In regards to the infant’s full stomach status secondary to pyloric stenosis, a study by Cook-Sather et al., comparing awake and rapid sequence induction, showed comparable results with awake intubation, rapid sequence induction, and modified rapid sequence induction [15]. Halogenated agents may be used for anesthesia maintenance; non-depolarizing muscle relaxants may be needed for adequate muscle relaxation. Nitrous oxide should be avoided, as it can cause bowel distention. Extubation should be attempted once the baby meets the standard extubation criteria. Local infiltration/transverse abdominis plane block avoids opioid analgesics for post-operative pain relief. Rectus sheath block is useful in a circum-umbilical incision, for open-pyloromyotomy. Ketorolac is not ideally indicated for use under the age of one.

There have been cases of episodes of apnea post-pyloromyotomy, hence it’s essential to monitor the baby in the intensive care unit (ICU) postoperatively for desaturations and bradycardia. Our patient was monitored for apnea owing to the presence of risk factors, such as anemia, post-conceptual age <60 weeks. In the setting of hereditary spherocytosis, it's likely that our patient may have multiple anesthetic exposures in the future, hence total intravenous anesthesia could have been a better alternative for inhalational anesthesia, to avoid neurotoxicity. However, no literature is available to support or refute the same.

## Conclusions

The anesthetic management of neonates with anemia, hereditary spherocytosis, hyperbilirubinemia, and pyloric stenosis is rare. It’s essential for one to know the physiological disturbances, complications, and consequences of the medical conditions as a puzzle in order to safely provide anesthesia for emergency surgeries with multiple comorbidities.

## References

[1] Tan AW, Leung P, Patil UP (2018). Spherocytosis in a Hispanic newborn presenting as early onset severe hyperbilirubinemia. Fetal Pediatr Pathol.

[2] Oprea AD (2012). Hematologic disorders. Stoelting's Anesthesia and Co-existing Disease.

[3] Narla J, Mohandas N (2017). Red cell membrane disorders. Int J Lab Hematol.

[4] Agre P, Orringer EP, Bennett V (1982). Deficient red-cell spectrin in severe, recessively inherited spherocytosis. N Engl J Med.

[5] Hankø E, Hansen TW, Almaas R (2005). Bilirubin induces apoptosis and necrosis in human NT2-N neurons. Pediatr Res.

[6] Bolton-Maggs PH (2004). Hereditary spherocytosis; new guidelines. Arch Dis Child.

[7] Ghoti H, Fibach E, Dana M (2011). Oxidative stress contributes to hemolysis in hereditary spherocytosis. Ann Hematol.

[8] Hevesi Z, Hannaman M (2012). Hyperbilirubinemia. Stoelting’s Anesthesia and Co-existing Disease.

[9] Wayne C, Hung JH, Chan E, Sedgwick I, Bass J, Nasr A (2016). Formula-feeding and hypertrophic pyloric stenosis: is there an association? A case-control study. Pediatr Surg.

[10] Michelle WD, Thomas JM (2018). Pediatric diseases: pyloric stenosis. Stoeltings Co-existing Diseases.

[11] Charles JC (2014). Pyloric stenosis. Miller’s Anesthesia.

[12] Kamata MR, Cartabuke JD, Tobias JD (2015). Perioperative care of infants with pyloric stenosis. Pediatr Anesth.

[13] Cook-Sather SD, Liacouras CA, Previte J, Markakis DA, Schreiner MS (1997). Gastric fluid measurement by blind aspiration in paediatric patients: a gastroscopic evaluation. Can J Anesth.

[14] Scrimgeour GE, Leather NW, Perry RS, Perry RS, Pappachan JV, Baldock AJ (2020). Gas induction for pyloromyotomy. Pediatr Anest.

[15] Cook-Sather SD, Tulloch HV, Cnaan A (1998). A comparison of awake versus paralyzed tracheal intubation for infants with pyloric stenosis. Anesth Analg.

